# Tissue inhibitor of metalloproteinases-3 mediates the death of immature oligodendrocytes via TNF-α/TACE in focal cerebral ischemia in mice

**DOI:** 10.1186/1742-2094-8-108

**Published:** 2011-08-29

**Authors:** Yi Yang, Fakhreya Y Jalal, Jeffrey F Thompson, Espen J Walker, Eduardo Candelario-Jalil, Lu Li, Ross R Reichard, Chi Ben, Qing-Xiang Sang, Lee Anna Cunningham, Gary A Rosenberg

**Affiliations:** 1 Department of Neurology, University of New Mexico Health Sciences Center, Albuquerque, NM 87131, USA; 2 Department of Neurosciences, University of New Mexico Health Sciences Center, Albuquerque, NM 87131, USA; 3 Department of Cell Biology and Physiology, University of New Mexico Health Sciences Center, Albuquerque, NM 87131, USA; 4 Department of Pathology, University of New Mexico Health Sciences Center, Albuquerque, NM 87131, USA; 5 Department of Anesthesia, University of California, San Francisco, San Francisco, CA 94110, USA; 6 Department of Chemistry and Biochemistry, Florida State University, Tallahassee, FL 32306, USA

## Abstract

**Background and Purpose:**

Oligodendrocyte (OL) death is important in focal cerebral ischemia. TIMP-3 promotes apoptosis in ischemic neurons by inhibiting proteolysis of TNF-α superfamily of death receptors. Since OLs undergo apoptosis during ischemia, we hypothesized that TIMP-3 contributes to OL death.

**Methods:**

Middle cerebral artery occlusion (MCAO) was induced in *Timp-3 *knockout (KO) and wild type (WT) mice with 24 or 72 h of reperfusion. Cell death in white matter was investigated by stereology and TUNEL. Mature or immature OLs were identified using antibodies against glutathione *S*-transferase-π (GST-π) and galactocerebroside (GalC), respectively. Expression and level of proteins were examined using immunohistochemistry and immunoblotting. Protein activities were determined using a FRET peptide.

**Results:**

Loss of OL-like cells was detected at 72 h only in WT ischemic white matter where TUNEL showed greater cell death. TIMP-3 expression was increased in WT reactive astrocytes. GST-π was reduced in ischemic white matter of WT mice compared with WT shams with no difference between KO and WT at 72 h. GalC level was significantly increased in both KO and WT ischemic white matter at 72 h. However, the increase in GalC in KO mice was significantly higher than WT; most TUNEL-positive cells in ischemic white matter expressed GalC, suggesting TIMP-3 deficiency protects the immature OLs from apoptosis. There were significantly higher levels of cleaved caspase-3 at 72 h in WT white matter than in KO. Greater expression of MMP-3 and -9 was seen in reactive astrocytes and/or microglia/macrophages in WT at 72 h. We found more microglia/macrophages in WT than in KO, which were the predominant source of increased TNF-α detected in the ischemic white matter. TACE activity was significantly increased in ischemic WT white matter, which was expressed in active microglia/macrophages and OLs.

**Conclusions:**

Our results suggested that focal ischemia leads to proliferation of immature OLs in white matter and that TIMP-3 contributes to a caspase-3-dependent immature OL death via TNF-α-mediated neuroinflammation. Future studies will be needed to delineate the role of MMP-3 and MMP-9 that were increased in the *Timp-3 *wild type.

## Background

Oligodendrocytes (OLs) undergo a complex pattern of death in cerebral ischemia with an early loss from glutamate excitotoxicity and a slower death due to apoptosis [1-4]. The vulnerability of OLs to ischemia was shown morphologically [5] and the molecular mechanism was related to excitotoxicity via the glutamate AMPA receptors [6,7]. Ischemia results in apoptosis of OLs, but the mechanisms involved are less well understood. The pro-inflammatory factor tumor necrosis factor-α (TNF-α) and TNF death receptors play an important role in OL death as shown *in vitro *and *in vivo *[1,8,9]. Cerebral ischemia results in an inflammatory response consisting of microglia activation, gliosis, and cell death. Activated microglia and reactive astrocytes produce TNF-α and interleukin-1β (IL-1β), which are associated with periventricular white matter damage in hypoxic neonatal brain [10]. Extravasation of inflammatory cells into the central nervous system (CNS) is facilitated by extracellular activities of matrix metalloproteinases (MMPs) that are regulated, in part, by the endogenous tissue inhibitors of metalloproteinases (TIMPs) [11].

Cell surface sheddases, including TNF-α converting enzyme (TACE) and stromelysin-1 (MMP-3) regulate the TNF superfamily of death receptors by activating the ligands and removing the death receptors from the cell surface. Tissue inhibitor of metalloproteinases-3 (TIMP-3) plays a central role in this process by inhibiting TACE and MMP-3. In an early study, TIMP-3 blocked the release of the TNF death receptor by TACE, promoting apoptosis [12]. More recently, we observed that TIMP-3 expression in ischemic neurons was associated with APO-1 (Fas/CD95) and DNA fragmentation [13]. Using neuronal cell cultures, we have demonstrated that TIMP-3 promoted apoptosis by preventing the shedding of Fas receptors from the neuronal cell surface during oxygen-glucose deprivation, and that the *Timp-3 *knockout (KO) mouse showed reduced neuronal death after a middle cerebral artery occlusion (MCAO) with reperfusion compared to the wild type (WT) [14,15].

Because of TIMP-3's unique role as a cell surface inhibitor of TACE and MMP-3, it could contribute to cell death in OLs by promoting retention of TNF death receptors [16]. To investigate the role of TIMP-3 in OL cell loss, we induced a middle cerebral artery occlusion (MCAO) in *Timp-3 *knockout (KO) and wild type (WT) mice followed by 24 and 72 h of reperfusion. We hypothesized that TIMP-3 and Fas/CD95 expression occurred early in neurons and preceded DNA fragmentation, but that in white matter TIMP-3 may contribute to OL death that is associated with TNF-α receptors. We report that TIMP-3 deficiency protects the immature OLs from apoptotic death in white matter induced by ischemia-reperfusion injury. The immature OL death in the WT was associated with increased expression of MMPs, TIMP-3, TNF-α/TACE in reactive astrocytes and/or infiltrating macrophages/microglia in white matter.

## Methods

### MCAO with reperfusion

The study was approved by the University of New Mexico Animal Care Committee and conformed to the National Institutes of Health Guide for the Care and Use of Animals in research. Eighty-nine male *Timp-3 *KO (n = 44) and WT (n = 45) mice weighing 20-30 g (age 10-12 weeks) were employed in this study. Fifty-nine animals were subjected to 90 min MCAO using the intraluminal thread method followed by 24 or 72 h reperfusion. MCAO was performed as previously described [15,17]. Mice underwent reperfusion via the circle of Willis. Thirty sham-operated animals were anesthetized and had a suture introduced into the common carotid artery and immediately removed.

### Primary antibodies and dilutions used in immunohistochemistry

Glial fibrillary acidic protein (GFAP) (1:400; Sigma), Iba-1 (1:200; Wako Pure Chemical Industries), GST-π (1:600, Abcam), GalC (1:300, Millipore), Fas (1:400; Santa Cruz Biotechnology), Fas ligand (1:400; Santa Cruz Biotechnology), MMP-2 (1:250; Chemicon), MMP-3 (1:1500; Chemicon), MMP-9 (1:1000; Chemicon), caspase-3 (1:1000, Cell Signaling), TIMP-3 (1:1000; Chemicon), TNF-α (15 μg/ml; R&D Systems), TNF Receptor I (1:1000; Abcam), von Willebrand Factor (vWF) (10 μg/ml; Chemicon), myeloperoxidase (MPO; 1:250; Santa Cruz Biotechnology), and several markers for oligodendroglia; RIP (1:200,000; Chemicon), CNPase (15 μg/ml; Chemicon), and CC-1 (APC; 10 μg/ml; Calbiochem).

### Immunohistochemistry

Brain tissues fixed with 2% paraformaldehyde, 0.1 M sodium periodate, 0.075 M lysine in 100 mM phosphate buffer at pH 7.3 (PLP) were used for immunohistochemical analysis. For immunofluorescence, 10 μm PLP-fixed brain sections were treated with acetone and blocked with 5% normal serum. Primary antibodies were incubated for two nights at 4°C. Slides were incubated for 90 min at room temperature with secondary antibodies conjugated with FITC or Cy-3 (Jackson). 4'-6-diamidino-2-phenylindole (DAPI; Molecular Probes) was used to label cell nuclei.

For brightfield immunolabeling, 10 μm PLP-fixed sections were rehydrated through alcohol series and incubated in phosphate buffered saline and 0.1% Tween-20 pH 7.3 (PBT) prior to blocking with 5% normal serum in PBT. Slides were incubated with primary antibodies for 48 h at 4°C followed by 1 h incubation with biotinylated secondary antibodies, Vectastain Elite ABC reagents (Vector Laboratories) and reacted with 3,3'-diaminobenzidine (DAB) was used for visualization of immunolabeling. Cresyl violet acetate (CVA, Sigma-Aldrich) was used for counterstaining.

Immunohistochemistry negative controls were incubated without the primary antibody or with normal (non-immune) IgGs and did not exhibit specific immunolabelling. All immunohistochemically stained slides were viewed on an Olympus BX-51 bright field and fluorescence microscope (Olympus America Inc.) equipped with an Optronics digital camera and Picture Frame image capture software (Optronics). Dual immunofluorescence slides were also imaged using high resolution confocal microscopy to verify co-localization of antigens (Zeiss LSM 510, Carl Zeiss Microimaging). Immunofluorescence was quantified using ImageJ software (NIH software).

### Stereological Counts

The cellular composition of the white matter is comprised of > 90% OLs [18] consisting of OL progenitors, immature, and mature OLs. Antibodies used to identify mature OLs, including CC-1, RIP, and CNPase, either labeled too few OL cell bodies or obscured the cell bodies in the processes. To measure white matter damage in mouse focal ischemia, we first assessed loss of OL-like cells in white matter [2] using multiple-antibody immunostaining [19]. Labeling cells with a Nissl substance stain, such as cresyl violet acetate (CVA), all cells are clearly observable and an assessment of OL density can be determined stereologically. To distinguish OLs from astrocytes and microglia, we stained slides with GFAP for astrocytes and Iba-1 for microglia, using the DAB method. All slides were counterstained with CVA. By eliminating DAB-stained astrocytes and microglia, small CVA-positive cell bodies that were negative for GFAP and Iba-1 immunoreactivity were identified as OL-like cells and quantified using unbiased stereology with StereoInvestigator (Version 6, MBF Bioscience) software controlling a motorized stage equipped Olympus BX-51 microscope [19]. Employing the optical fractionator function of StereoInvestigator, we estimated OL-like cell number in the medial corpus callosum (CC) and external capsule (EC) regions of white matter over a total distance of 3.0 mm rostral and caudal to bregma. Tissue sections were blinded to the investigator as to animal identity and reperfusion time. A subset of slides was stained with myeloperoxidase or von Willebrand factor to identify neutrophils and endothelial cells. These could be distinguished from the OL-like cells based on morphology (data not shown).

### Western blots

Western blot was performed to determine protein levels in ischemic white matter. Proteins were extracted in RIPA buffer from punch biopsies of white matter in ischemic and nonischemic external capsule regions of *Timp-3 *KO and WT mice. 50 μg total protein was separated on 10% or 12% gels. The proteins were transferred to polyvinylidene fluoride (PVDF) membranes. The membranes were then incubated with primary antibodies: MBP (1:2000, Millipore), GST-π (1:5000, Abcam), GalC (1:500, Millipore), MMP-3 (1:1000, Epitomics), both inactive (pro-) and active (cleaved) caspase-3 (1:1000; Cell Signaling Technology), and TNFR1 (1:1000; Abcam). The membranes were incubated with the respective secondary antibodies and blots were developed using the West Pico Detection System (Pierce). Protein bands were visualized on X-ray film. Semiquantitation of target protein intensities was done with the use of Scion image software (Scion), and Brilliant Blue R (Sigma-Aldrich) staining on the same PVDF membranes was used to normalize protein loading and transfer. The results are reported as normalized band intensity for quantifying relative protein expression.

### TUNEL assay

Terminal deoxynucleotidyl transferase-mediated dUTP nick end labeling (TUNEL) assay was done to identify the extent of DNA fragmentation. Using the NeuroTACS II kit (Trevigen) brain sections were treated following the procedure specified by the manufacturer. For dual immunofluorescence, the brain sections were then exposed to the primary antibodies and streptavidin fluorescein and Cy-3 secondary antibodies. For positive controls the nuclease provided with the kit was added to the labeling reaction mix and induced TUNEL positivity in all cells. As negative controls, slides were prepared in a labeling reaction mix without the TdT enzyme resulting in no TUNEL positivity (data not shown).

### Enzymatic assay of MMP-3 activity using a fluorescence resonance energy transfer (FRET) peptide

MMP-3 enzymatic activity was assessed using a peptide-based FRET assay. In the intact FRET peptide, Mca-Arg-Pro-Lys-Pro-Val-Glu-Nva-Trp-Arg-Lys(Dnp)-NH_2_, obtained from Bachem (Torrance, CA) the fluorescence of Mca (7-methoxycoumarin-4-acetyl) is quenched by Dnp (2,4-dinitrophenyl). Upon cleavage into two separate fragments by the MMP-3 present in the sample, the fluorescence of Mca was recovered, and monitored at λ_ex _= 328 nm and λ_em_= 393 nm using a spectrofluorimeter (Perkin-Elmer LS 50B).

Brain tissue was homogenized in lysis buffer containing 50 mM Tris-HCl pH 7.6, 150 mM NaCl, 5 mM CaCl_2_, 0.05% Brij-35, 0.02% NaN_3_, and 1% Triton X-100, and centrifuged at 12,000 × *g *for 5 min at 4°C. Equal amount of total protein (6-10 μg) was mixed with assay buffer containing 50 mM HEPES buffer pH 7.5, 200 mM NaCl, 10 mM CaCl_2_, 0.01% Brij-35, and fluorogenic substrate (1 μM final concentration) to a final volume of 200 μl. Fluorescence was monitored for 15 min. The relationship between fluorescence units and nM of product produced was determined from the fluorescence values obtained when all the substrate was hydrolyzed.

### Fluorometric assay of TNF-α converting enzyme (TACE) activity

TACE activity was measured using the fluorometric SensoLyte 520 TACE (α-Secretase) Activity Assay Kit (AnaSpec) according to the manufacturer's instructions. SensoLyte™ 520 TACE Activity Assay Kit uses a 5-FAM (fluorophore) and QXL™ 520 (quencher) labeled FRET peptide substrate for continuous measurement of enzyme activity. In the intact FRET peptide, the fluorescence of 5-FAM is quenched by QXL™ 520. Upon cleavage of the FRET peptide by the active enzyme, the fluorescence of 5-FAM is recovered, and can be continuously monitored at λ_ex _= 490 nm/520 nm. TACE activity was measured in the brain tissue homogenized as described before for MMP-3 activity. Equal amount of total protein was mixed with assay buffer (AnaSpec) and TACE substrate to a final volume of 100 μl. Fluorescence was monitored for 60 min with data recorded every 5 min. Fluorescence was expressed as relative fluorescence units (RFU). Some brain samples from KO and WT mice had 10 μl of 10 μM TACE inhibitor, TAPI-0, added as a control.

### Data analysis

Statistical comparisons among groups were done using ANOVA with post-hoc analysis for multiple *t*- tests (Prism 4.0, GraphPad Software Inc.). All data are presented as mean ± standard error of the mean (S.E.M.). Statistical significance was set at *p *< 0.05.

## Results

### *Timp-3 *KO mice were protected from delayed immature OL death

**Figure 1:**
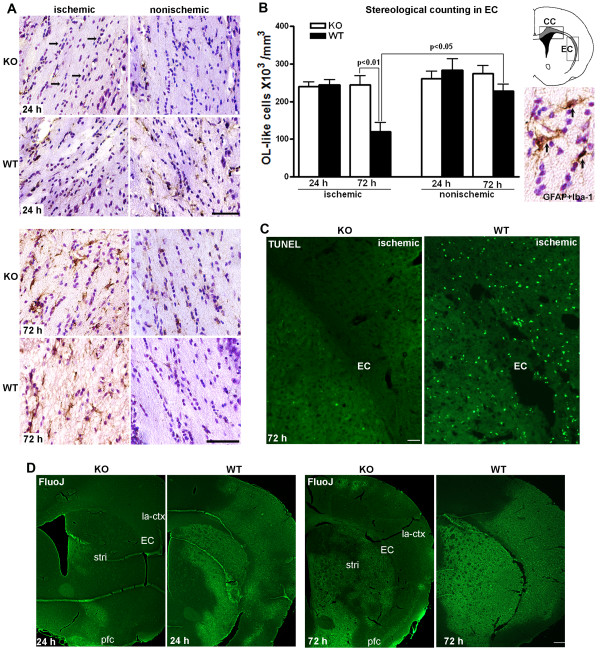
**White matter injury and OL-like cell loss in EC at 72 h in *Timp-3 *KO and WT mice**. **A**. Representative regions from the nonischemic and the ischemic EC at 24 and 72 h. Iba-1 (microglia/macrophage) and GFAP (astrocyte) were both stained with DAB and counterstained with cresyl violet acetate (CVA). The intact linear rows of dark-staining nuclei by CVA were formed by OL-like cells (arrows) in nonischemic ECs at 24 and 72 h, as well as in ischemic ECs at 24 h. Ischemic EC shows massive DAB-positive glial cells at 72 h. A marked loss of CVA positive cells was seen in ischemic EC in WT mouse compared with that in KO mouse. Scale bars = 50 μm. **B**. Stereological counting of OL-like cells in ischemic EC at 24 and 72 h reperfusion shows OL-like counts were similar at 24 h between *Timp-3 *KO and WT ischemic. A significant loss of OL-like cells occurred at 72 h in the WT ischemic EC compared to KO ischemic and to the WT nonischemic EC (p < 0.01 and 0.05, respectively, n = 6). From 24 to 72 h, OLs in ischemic KO EC remained unchanged. The brain cartoon shows location of regions used for OL counting (shaded areas). CC: corpus callosum; EC: external capsule. Micrographic image shows Iba-1 and GFAP (arrows) visualized by DAB in higher magnification. **C**. TUNEL assay demonstrates apoptotic cell death in ischemic EC at 72 h reperfusion. TUNEL-positive cells were seen in ischemic WT EC. Scale bar = 100 μm. **D**. Distribution of neuronal degeneration in ischemic hemispheres in *Timp-3 *KO and WT mice at 24 and 72 h of reperfusion following 90 min transient MCAO. Coronal sections were stained for degenerating neurons using Fluoro-Jade (FluoJ). Number of FluoJ-positive cells was much greater in WT than in KO at both tiom points. La-ctx: lateral cortex, stri: striatum, pcf: piriform cortex. Scale bar = 100 μm.

Two regions of ischemic white matter were analyzed: the corpus callosum (CC) and the external capsule (EC). No OL-like cell loss was seen in ischemic CCs in both KO and WT mice at 24 and 72 h (data not shown) or in ischemic EC at 24 h (Figure 1A, top two panels). Representative regions from the nonischemic and the ischemic EC at 72 h showed the normal appearing OL-like cells in the nonischemic sides and the reactive astrocytosis and/or microglia/macrophages in the ischemic ECs in KO and WT (Figure 1A. bottom two panels). The ischemic EC in KO showed no observable loss of OL-like cells, while the ischemic EC in WT had marked loss of OL-like cells (Figure 1A, bottom two panels).

Next, the loss of OL-like cells in ischemic white matter was quantified by unbiased stereology. At 24 h, KO and WT mice showed similar cell counts in the ischemic and the nonischemic hemispheres (Figure 1B). By 72 h, there was a significant loss of OL-like cells in the ischemic EC of WT compared to KO mice (p < 0.01). Furthermore, there was a significant loss of cells in the ischemic compared to the nonischemic hemisphere in WT only (p < 0.05). In the CC, no significant reduction of OL-like cells was detected in either WT or KO ischemic CC at 72 h (data not shown). Next, we investigated apoptotic cell death in ischemic EC at 72 h of reperfusion by TUNEL assay. More TUNEL-positive cells were detected in ischemic WT hemisphere including the EC region compared with KO (Figure 1C). The difference in the distribution of TUNEL-positive cells between the ischemic KO and WT hemispheres is consistent with the patterns of the neuronal degeneration revealed by Fluoro-Jade staining (Figure 1D). This result suggested that ischemia-reperfusion injury induced a delayed cell loss in white matter in which TIMP-3 may be involved.

**Figure 2:**
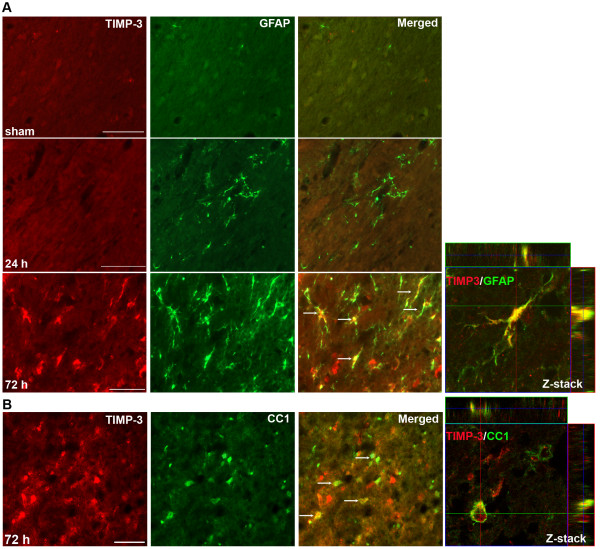
**TIMP-3 expression in astrocytes and OLs in ischemic EC at 72 h in *Timp-3 *WT mice**. **A**. Photomicrographs show double immunofluorescent-labeling of TIMP-3 and GFAP in sham-operated and ischemic EC at 24 and 72 h of reperfusion. TIMP-3 was not expressed in GFAP-labeled astrocytes at 24 h, but by 72 h TIMP-3 was markedly expressed and co-localized with astrocytes (arrows). The co-localization of TIMP-3 and GFAP was confirmed by Z-stack confocal image. **B**. CC1-positive OLs express TIMP-3 (arrows) in ischemic EC at 72 h. The co-localization of TIMP-3 and CC1 was confirmed by Z-stack confocal image. Scale bars = 50 μm.

We then studied the expression of TIMP-3 in astrocytes and OLs in WT mice. At 24 h, very little TIMP-3 was seen in the white matter. We observed a new population of GFAP-positive astrocytes closely surrounding the core infarct areas at 72 h (data not shown), which has been reported by others [20]. Maximal TIMP-3 expression occurred at 72 h in GFAP-positive astrocytes in ischemic EC (Figure 2A). Most of the astrocytes were co-labeled with TIMP-3 and co-localization of TIMP-3 with CC1, an OL marker, was also seen by 72 h (Figure 2B). This temporal and spatial relationship of TIMP-3 expression and the loss of OL-like cells in ischemic EC indicated the involvement of TIMP-3 in the white matter damage in focal cerebral ischemia.

**Figure 3:**
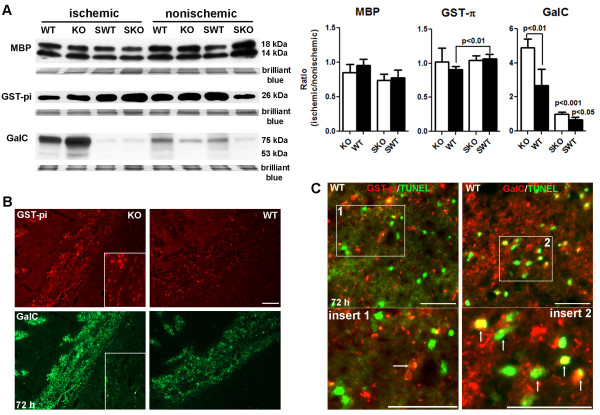
**Immature and mature OL loss in *Timp-3 *WT mice at 72 h**. **A**. Western blots for MBP, GST-pi (π) and GalC in white matter in reperfusion injury at 72 h in WT and KO mice. No significant changes of MBP level were detected at 72 h reperfusion after ischemia in both WT and KO mice. Significantly decreased levels of GST-π was seen in ischemic WT compared with sham WT (SWT) (p < 0.01, n = 5 for each group). Significant increase of GalC was seen in ischemic WT and KO compared with sham WT (*p *< 0.001, n = 5 for each group) and sham KO (SKO) (*p *< 0.05), respectively. *Timp-3 *KO mouse shows significantly higher levels of GalC in white matter than WT mouse (*p *< 0.01). Intensity of total proteins stained by Brilliant blue R on the same blots was performed as control for protein loading and transfer normalization. The results were presented as ratio of protein expression between ischemic and nonischemic white matter. **B**. Double-immunostaining of GST-π (red) and GalC (green), showing the expression of mature and immature OLs in ischemic external capsule at 72 h reperfusion in *Timp-3 *KO and WT mice. More GST-π- and GalC-positive signals were seen in KO ischemic EC than in WT. Inserts show immunohistochemistry of GST-π and GalC in sham-operated animals. **C**. Co-localizarion of TUNEL labeling GST-π or GalC immunohistochemistry in ischemic WT EC at 72 h reperfusion. Arrows show the co-localization of TUNEL-positive cells with GST-π or GalC expression was seen in ischemic EC (insert 1 and 2). Scale bars = 50 μm.

To further investigate the role of TIMP-3 in OL death, we examined the expression of myelin basic protein (MBP) and glutathione *S*-transferase π (GST-π, or GST-pi) [21], markers of mature OL, in ischemic ECs of KO and WT mice at 72 h using Western blot analysis (Figure 3A). Unexpectedly, we found no significant changes of MBP level either in ischemic ECs between KO and WT mice or between ischemic ECs and sham ECs. However, we observed a significant decrease of GST-π level in WT ischemic EC compared with sham WT mice. Next, we tested the expression of galactocerebroside (GalC), a marker of immature OL [22,23]. We found that a significant increase in GalC was seen in ischemic ECs of WT and KO by 72 h reperfusion after ischemia compared with sham WT (*p *< 0.001) and sham KO (*p *< 0.05), respectively. Most importantly, TIMP-3 KO mouse shows significantly higher levels of GalC expression in white matter than WT mouse (*p *< 0.01). These findings suggested that TIMP-3-mediated delayed OL death mainly occurs in immature OLs in white matter after ischemic stroke with reperfusion injury.

To confirm the observation that TIMP-3 contributed to delayed immature OL death in ischemic white matter, we next performed immunohistochemistry of GST-π and GalC on brain tissue from KO and WT mice with 90 min-MCAO and 72 h reperfusion (Figure 3B). Double-immunostaining of GST-π and GalC demonstrated stronger immunohistochemical signals of GST-π and GalC in ischemic KO EC than WT EC. Furthermore, we detected apoptotic death using TUNEL assay in OLs. We found TUNEL-positive cells in ischemic EC at 72 h, which mostly co-localized with GalC-positive OLs (Figure 3C).

### Caspase-3 increased in WT compared to *Timp-3 *KO

**Figure 4:**
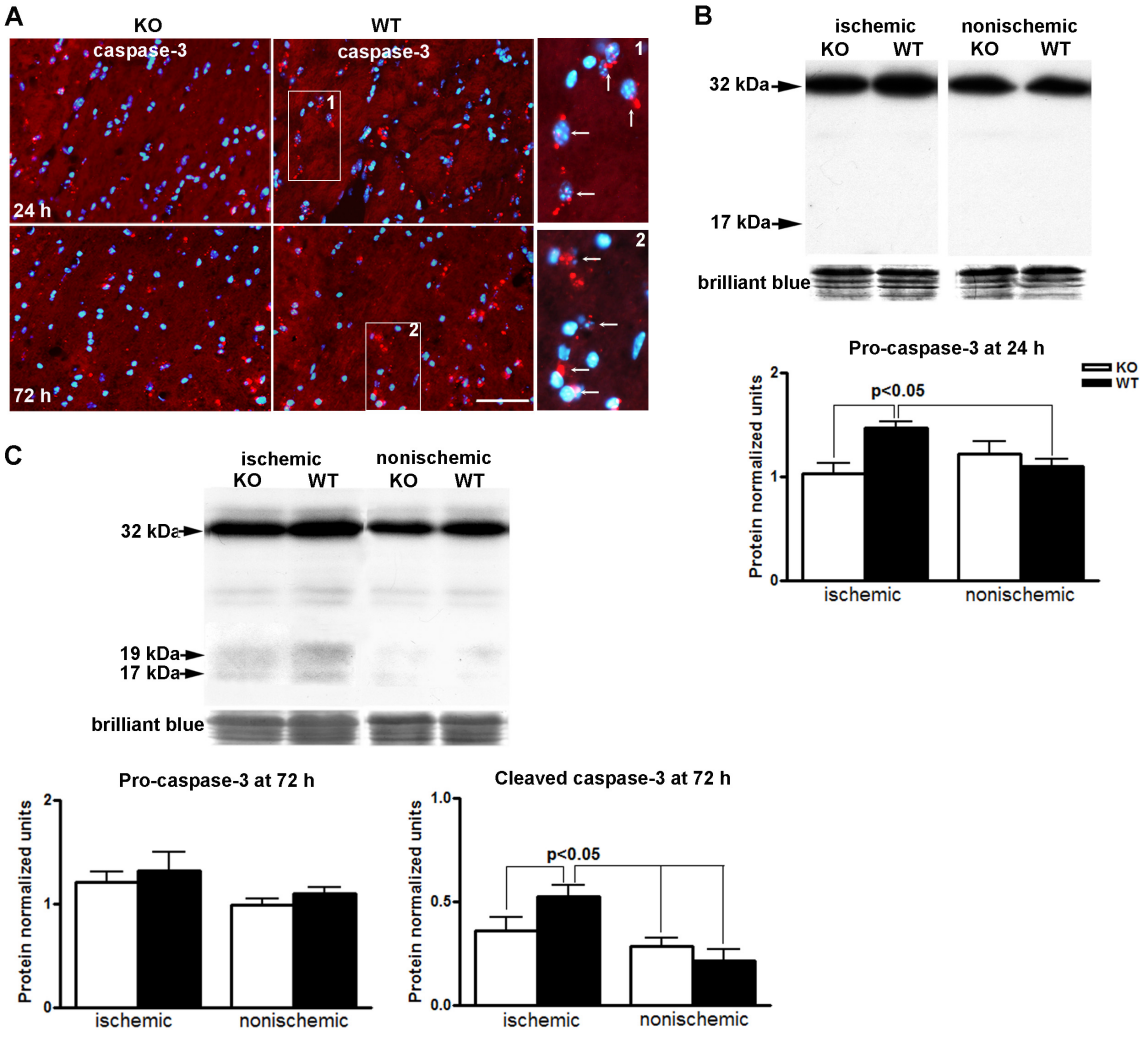
**Caspase-3 expression in ischemic EC in *Timp-3 *KO and WT mice at 24 and 72 h**. **A**. Immunostaining shows that expression of caspase-3 was detected in ischemic WT EC 24 h after reperfusion. The increased caspase-3 expression in WT was present in OL-like cells. Less caspase-3 immunostaining was detected in both ischemic WT and KO ECs at 72 h, while nuclear counterstaining with DAPI (blue) showed greater loss of cells in ischemic WT EC. Insert 1 and 2 show caspase-3 immunostaing is associated with nuclei (arrows). Scale bars = 50 μm. **B**. Western blots for expression of caspase-3 in white matter at 24 h in *Timp-3 *KO and WT mice. The graph shows an increase in pro-caspase-3 band at 32 kDa in the ischemic EC of the WT mice (p < 0.05, n = 4). Cleaved caspase-3 (17 kDa and 19 kDa) was not detected. Brilliant blue R staining on the same blots was performed as control for protein loading and transfer. **C**. Western blots for caspase-3 in white matter in reperfusion injury at 72 h showed no difference in expression of pro-caspase-3 between WT and KO, but increased cleaved caspase-3 (19 kDa and 17 kDa) in ischemic WT white matter (p < 0.05, n = 6 for each group).

To study the possible mechanism of TIMP-3-mediated cell death, we used immunohistochemistry to determine the expression of caspase-3, since apoptosis has been implied in immature and mature OL death [1,10,24-26]. In the ischemic EC of the WT, greater caspase-3 immunoreactivity was detected at 24 h of reperfusion than in the KO. There was loss of DAPI stained nuclei (blue) in the WT EC as compared with the KO EC at 72 h reperfusion (Figure 4A).

Western blots were done with an antibody that recognizes both pro- and cleaved-caspase-3. The activated caspase-3 often appears as multiple cleaved bands migrating variably in the 17-20 kDa range [27]. There was no detectable cleaved (active) caspase-3 (17/19 kDa) in white matter of KO and WT mice at 24 h after stroke. Western blots exhibited strong immunoreactive bands at 32 kDa corresponding to the pro- (inactive) caspase-3 (Figure 4B). A significant increase of pro-caspase-3 was demonstrated in ischemic white matter of WT mice (p < 0.05), indicating that caspase-3 was markedly induced in WT mice at 24 h reperfusion after stroke. By 72 h there were no differences in pro-caspase-3 expression in the white matter of KO or WT mice (Figure 4C). More importantly, a statistically increased level of cleaved caspase-3 (17/19 kDa) was seen in ischemic WT white matter (p < 0.05), implicating an apoptotic mechanism in OL loss (Figure 4C).

### MMP-3 and -9 increased in WT compared to *Timp-3 *KO

**Figure 5:**
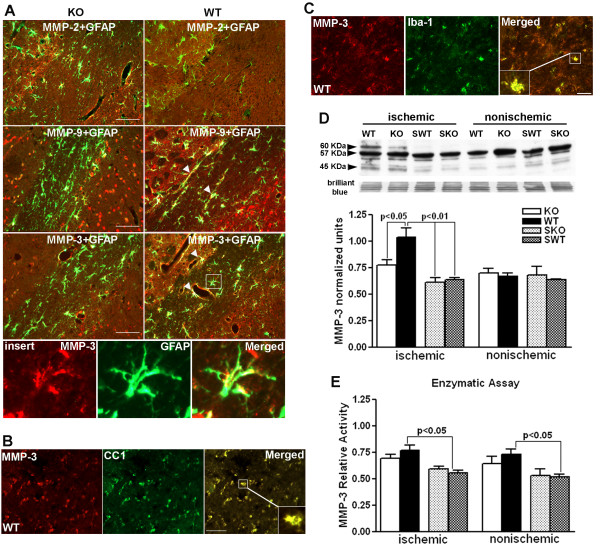
**Expression of MMPs in ischemic EC in *Timp-3 *KO and WT mice at 72 h**. **A**. Immunohistochemistry for MMPs in ischemic external capsule (EC) at 72 h reperfusion in *Timp-3 *KO and WT mice. The *Timp-3 *knockout had increased MMP-2 (red) immunoreactivity in ischemia, which co-localized with GFAP-expressing astrocytes (yellow). MMP-9 and MMP-3 were co-localized with astrocytes in the WT. Arrowheads show MMP-9 and -3 in astrocytes around vessels. Insert shows co-localization of MMP-3 and GFAP-positive astrocyte. Scale bar = 100 μm. **B**. Immunostaining shows MMP-3 expression in CC1-positive OLs in ischemic WT EC. **C**. Immunostaining shows MMP-3 expression in Iba-1 positive microglia/macrophages. Scale bar = 50 μm. **D**. Western blot of MMP-3 at 72 h reperfusion in *Timp-3 *KO and WT mouse brains. Western blots of MMP-3 standard and mouse brain tissues show the proform of MMP-3 at 57 kDa and an active form at 45 kDa. A 60 kDa glycosylated form of MMP-3 is also present in all brain samples. Brilliant blue R staining on the same blots was performed as control for protein loading and transfer. Quantification of pro and active MMP-3 forms together showed a significant increase in MMP-3 (57 kDa and 45 kDa) in ischemic WT compared with ischemic KO, sham KO and WT and nonischemic KO and WT (p < 0.05, n = 6 for each group). No significant difference was found between ischemic and nonischemic hemispheres in the KO. **E**. Fluorogenic substrate assay for MMP-3 activity shows significant increase in MMP-3 relative activity in both ischemic and nonischemic WT compared with sham WT (p < 0.05, n = 6 for each group).

Since TIMP-3 regulates the activities of MMPs that are important in processes involved in growth, cell death, and tissue repair [28], we next investigated the expression of MMP-2, -3 and -9 in the ischemic white matter of *Timp-3 *KO and WT utilizing immunohistochemistry (Figure 5A). Co-labeling of the MMPs and GFAP showed more MMP-2 immunoreactivity in the KO white matter than in the WT at 72 h. Increased expression of MMP-2 in *Timp-3 *KO mice had been observed previously in other disease models and attributed to chronic damage in heart and lung [29,30]. The increased MMP-2 expression was seen in GFAP-positive cells and around blood vessels. On the other hand, WT mice had more immunostaining for MMP-3 and -9 in EC than seen in the KO at 72 h. MMP-3 and-9 were seen in astrocytes around blood vessels (Figure 5A), while MMP-3 was also seen in OL cells and microglia/macrophages in the core infarcted white matter where very few astrocytes were detected (Figure 5A, B and 5C).

The observation of higher expression of MMP-3 in glial cells in WT white matter was unexpected since TIMP-3 is supposed to inhibit MMP-3 on the cell surface [28]. We quantified the MMP-3 protein in white matter by Western blot and measured its activity with a fluorogenic activity assay. We found increased MMP-3 (both pro- and active forms) in the ischemic hemisphere of the WT compared to the KO (Figure 5D). The fluorogenic assay showed a statistically significant increase in the activation of MMP-3 in the WT only (Figure 5E).

### Increased microglia/macrophage expressing TNF-α and TACE activity in WT compared to *Timp-3 *KO

**Figure 6:**
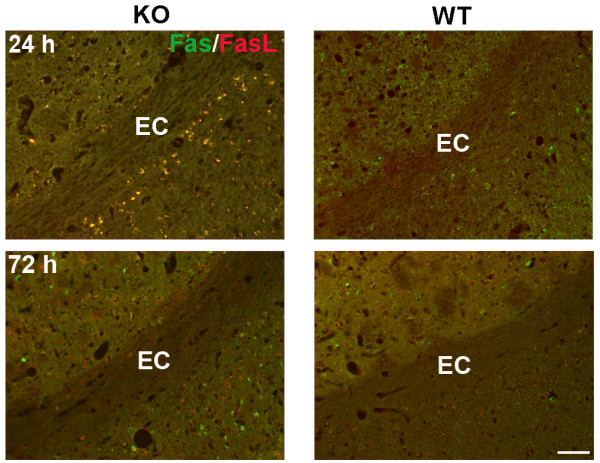
**A. Immunohistochemistry for Fas and FasL in ischemic EC at 24 and 72 h in *Timp-3 *KO and WT mouse brains**. No positive signal of Fas (green) and FasL (red) was detected in both KO and WT ECs. Scale bars = 50 μm.

**Figure 7:**
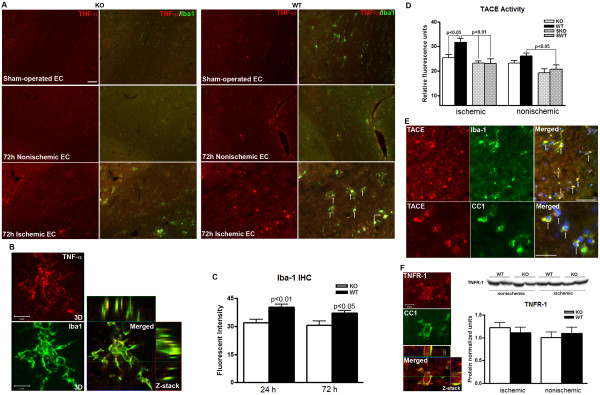
**Expression of TNF-α and TACE in ischemic EC in *Timp-3 *KO and WT mice at 72 h**. **A**. Double-staining of TNF-α (red) and Iba-1 (green), showing the expression of TNF-α in microglia/macrophages (Iba-1 positive cells) in ischemic EC at 72 h reperfusion in *Timp-3 *KO and WT mice. More TNF-α- and Iba-1-positive cells were seen in WT ischemic EC (arrows) than in KO. Scale bar = 40 μm. **B**. The three-dimension (3D) and Z-stack confocal images shows the co-localization of TNF-α in macrophage. Scale bar = 5 μm. **C**. Quantification of Iba-1 fluorescent intensity showed greater Iba-1-positive macrophages/microglia in *Timp-3 *WT mice compared with KO at both 24 and 72 h (p < 0.05, n = 5 for each group). **D**. TACE activity measured by a fluorometric assay in ischemic white matter at 72 h reperfusion in *Timp-3 *KO and WT mouse brains. Significant increase in TACE relative activity was seen in ischemic WT compared with ischemic KO (p < 0.05, n = 6) and sham WT (SWT) and KO (SKO) (p < 0.01). A greater level of TACE activity was seen in nonischemic WT compared with contralateral sham WT (p < 0.05). **E**. Top panel: Co-localization of TACE and Iba-1 in microglia/macrophages (arrows). Bottom: TACE and CC1 co-localization in OLs (arrows). Scale bars = 50 μm. **F**. Z-stack confocal images show TNFR-1 co-localized in CC1-positive OLs. Western blot analysis shows no significant changes ether between ischemic and nonischemic hemispheres or between KO and WT mice (n = 6).

Death of OLs can occur through death receptors of the TNF superfamily, including Fas/FasL and TNF-α/TNFR. Double immunostaining with Fas and FasL antibodies demonstrated greater Fas/FasL signal in the gray matter of KO mice at 24 h (Figure 6). Considering the neuronal degeneration occurs earlier and greater in WT gray matter than KO (Figure 1D), the greater Fas/FasL signal in gray matter in KO mice at 24 h suggests delayed cell death compared with WT mice. However, Fas/FasL signal was not detected in white matter in KO or WT mice at both 24 and 72 h reperfusion after focal ischemia (Figure 6). Since TNF-α and activated microglia and macrophages are associated with white matter damage, we then investigated the TNF-α expression in microglia/macrophages. TNF-α was increased in white matter of *Timp-3 *WT mice after 72 h reperfusion, and it was co-localized with Iba-1-positive microglia/macrophages (Figure 7A and 7B). Furthermore, we observed greater Iba-1-positive microglia/macrophages that expressed TNF-α in WT white matter at 72 h. For quantification of Iba-1, we evaluated fields of the ischemic EC as well as cortical and striatal tissue immediately bordering the EC in each section, quantifying the amount of fluorescent intensity of Iba-1 as assessed in five brains per group at each time point. We found significantly greater fluorescence of Iba-1 in ischemic WT EC compared to KO at both 24 and 72 h after ischemia (Figure 7C). These results suggest that TIMP-3 facilitates the inflammatory response to ischemia-reperfusion injury.

Since TIMP-3 inhibits TACE, which both generates soluble TNF-α (17 kDa) from the 21 kDa cell surface form and cleaves the TNF receptors, we measured TACE activity in white matter from *Timp-3 *KO and WT mice at 72 h reperfusion. Ischemia with 72 h reperfusion resulted in a significant increase in activation of TACE in white matter of WT mouse compared with KO (Figure 7D). The double staining of TACE and Iba-1 or CC1 showed the expression of TACE in Iba-1-positive microglia/macrophages and OLs in WT white matter (Figure 7E).

The excess presence of TNF-α in white matter could trigger cell apoptosis by ligation with the TNFR-1. We determined the presence of this receptor and the cell type expressing it by immunohistochemistry. We found that the TNFR-1 co-localized with the OL marker, CC1 (Figure 7F). Western blot showed no difference of TNFR-1 expression between KO and WT mice (Figure 7F).

## Discussion

We found that deletion of the *Timp-3 *gene reduced immature OL death at 72 h following MCAO-induced focal ischemia with reperfusion. TUNEL assay demonstrated apoptotic death of immature OLs. There was an increase in pro-caspase-3 at 24 h and cleaved caspase-3 at 72 h in white matter, indicating apoptosis was occurring at the time of delayed immature OL death. The presence of *Timp-3 *in the wild type led to an exaggerated inflammatory response with an increase in both infiltration of microglia/macrophages at 72 h and expression of MMP-3 and -9 in reactive astrocytes around blood vessels in the white matter. We found increased activity of TACE and TNF-α in microglia/macrophages, while TNFR-1 was expressed on OLs. Finally, WT mice had an increase in active microglia/macrophages compared with KO mice. These results provide the first evidence for TIMP-3 induction of inflammation and promotion of delayed immature OL death via a TNF-α-mediated mechanism.

An unexpected finding in this study was that TIMP-3 mainly promoted immature OL rather than mature OL death. Glutathione *S*-transferase π (GST-π) is a cytosolic isoenzyme in mature OLs, which is a marker of mature OLs in mammalian brain [21,31-33]. Western blot and immunohistochemistry showed a significant decrease in GST-π protein in the ischemic EC of *Timp-3 *WT mice. Although less GST-π protein disruption was seen in the KO, MBP expression was similar in WT and KO mice. We found preferential damage in immature OLs, which has been reported in perinatal hypoxia-ischemia [4]. Oligodendrocyte-specific galactosphingolipid (GalC) is expressed during differentiation of OL lineage cells, including OL progenitor cells (OPCs), premyelinating and myelinating OLs [34]. We used GalC as a marker for immature OLs [35]. Our data demonstrated a significant increase in GalC expression in ischemic EC in both KO and WT mice. We found that immature OLs undergo TIMP-3-mediated apoptosis associated with TIMP-3 expression in reactive astrocytes. These findings are consistent with the previous observation that OL progenitors are more susceptible to death due to hypoxia-ischemia than mature OLs in neonatal mice [25].

Cell surface receptors and ligands of the TNF-α superfamily play a fundamental role in apoptosis during neuroinflammation and overproduction of the proinflammatory cytokine, TNF-α, has been implicated in the pathogenesis of white matter damage [10,36]. TNF-α decreases the number of OL progenitors by causing their apoptosis [10,26,37]. Activated microglia and reactive astrocytes release TNF-α and other cytokines [38]. We observed an increased infiltration of TNF-α- and TACE-expressing microglia/macrophages in ischemic white matter of WT mouse at 72 h. *Timp-3 *KO mouse had reduced TNF-α expression and TACE activity and showed fewer microglia/macrophages in ischemic white matter, which is consistent with TIMP-3's role in enhancing inflammation in other tissues [39].

Co-labeling of MMPs and GFAP indicated astrocytes showed more MMP-2 immunoreactivity in the KO white matter than WT, which is consistent with earlier studies showing that *Timp-3 *KO mice have greater MMP-2 activation during physiological and pathological events [29,30]. On the contrary, WT mice showed more immunostaining for MMP-3 and MMP-9 in ischemic white matter than KO, and in reactive astrocytes around vessels. MMPs disrupt the blood-brain barrier by cleaving tight junction proteins at an early stage after ischemia-reperfusion injury in the rat [40]. MMP-3 can proteolytically activate MMP-9 [41]. Therefore, increased MMP-3 and -9 in astrocytes in white matter may play a role in BBB damage with facilitation of infiltration of macrophages in WT mouse. In addition, we also found increased active MMP-3 in OL-like cells and microglia/macrophages. MMP-3 is associated with the neuroinflammatory response. MMP-3 was shown to activate microglia and increase the neuroinflammatory response with expression of cytokines such as TNF-α [42]. In dopaminergic neurons MMP-3 may have an intracellular role in cell death [43,44]. We found increased MMP-3 (both pro- and active forms) in the ischemic hemisphere of the WT compared to the KO. A fluorogenic assay showed a significant increase in the activity of MMP-3 in the WT only. Although some studies suggest that MMP-3 contributes to cell death, MMP-3 was protective to neurons in culture exposed to doxorubicin through its role in cleaving the death receptors and ligands [15]. Studies with the *Mmp-3 *knockout mouse will be needed to resolve this issue.

OLs have cell surface receptors for members of the TNF-α death receptor family, including TNFR-1, TNFR-2, and Fas/CD95 [45]. TNF-R1 has an intracellular death domain and its activation leads to cell apoptosis [46]. Aberrant TNF-α/TNF-R1 signaling can have a potentially major role in the CNS pathologies in which OL apoptosis and demyelination are primary pathological features [47]. TNF-α is toxic to OLs in culture and in periventricular white matter of hypoxic rats [26,48]. TIMP-3 inhibits the action of TACE, which both activates TNF-α to the mature 17-kDa form and releases TNF death receptors from the cell surface [39]. Because TIMP-3 interacts with a number of cell surface processes, including formation of TNF-α by TACE, release of TNFR-1 from the cell surface, and other events, promoting either cell death or remodeling, the effect of TIMP-3 is difficult to predict [39].

**Figure 8:**
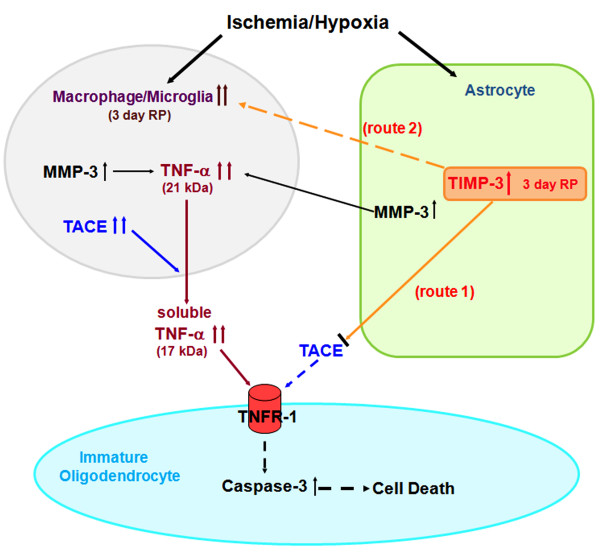
**Schematic drawing of hypothesis how TIMP-3 facilitates immature OL death after transient focal cerebral ischemia in mouse**. Route 1: TIMP-3 could block the shedding of various members of the death receptor families, including TNFR-1, by inhibiting TACE, stabilizing the death receptor on the surface of OLs. Route 2: Later (3 days reperfusion; RP), TIMP-3 enhances the inflammatory response and the release of inflammatory mediators in glial cells including TNF-α and MMP-3, which promote the OL death through a TNF-α-mediated mechanism.

It should be noted that the MCAO produces damage to the striatum, cortex, and the adjacent white matter. This could result in some of the molecules produced by the injury of the striatum/cortex influencing the damage in the white matter. Other mechanisms of cell death are possible in OLs where studies have shown that glutamate induces OL cell death both through an action on the cell body and on the ion channels of the axon [6,49,50]. While the action of TIMP-3 in OL death remains to be fully elucidated, it is possible to construct a hypothetical mechanism to explain TIMP-3 involvement in the OL death that involves the activation of microglia/macrophages by TIMP-3, which also inhibits shedding of TNFR-1 by TACE. Figure 8 shows a hypothetical mechanism for TIMP-3-facilitated death of immature OLs in transient cerebral focal ischemia in mice.

In conclusion, our results show that TIMP-3 contributes to immature OL death in ischemic white matter by increasing TACE, TNF-α, cleaved caspase- 3, and activated MMP-3 and -9. We observed a cellular preference with TIMP-3 strongly expressed in GFAP-positive astrocytes, while TNF-α, TACE and MMP-3 appear in microglia/macrophages and TNFR-1 is found in OLs. These results are the first to implicate TIMP-3 in the death of immature OLs by apoptosis and they suggest that an interaction occurs between astrocytes expressing TIMP-3, microglia/macrophages expressing TNF-α and MMP-3, and OLs expressing TNFR-1 that facilitates the death process. These results may have implications for the delayed death of vulnerable OLs seen in a number of hypoxic/ischemic neurological conditions.

## List of abbreviations

OL: oligodendrocyte; CC: corpus callosum; EC: external capsule; TIMP-3: Tissue inhibitor of metalloproteinases-3; MMPs: matrix metalloproteinases; TNF-α: tumor necrosis factor-α; MCAO: middle cerebral artery occlusion; KO: knockout; WT: wild type; GST-π (pi): glutathione *S*-transferase π (pi); GalC: galactocerebroside; TACE; TNF-α converting enzyme; TNFR-1: TNF Receptor I; GFAP: glial fibrillary acidic protein; DAPI: 4'-6-diamidino-2-phenylindole; DAB: 3,3'-diaminobenzidine; CVA: cresyl violet acetate; TUNEL: terminal deoxynucleotidyl transferase-mediated dUTP nick end labeling; FRET: fluorescence resonance energy transfer; BBB: blood-brain barrier; OPCs: oligodendrocyte progenitor cells.

## Competing interests

The authors declare that they have no competing interests.

## Authors' contributions

YY designed the experiments, carried out animal surgery and confocal microscopy, analyzed the data, and wrote the manuscript. YY, FYJ and JFT performed the biochemical and histologic studies. EJW, JFT and RRR carried out the stereological and morphological study. ECJ participated in the biochemical studies. BC and QXS performed the MMP-3 activity assay. LL and LAC helped with the MCAO model in mouse. GAR who is the leader of the group conceived and coordinated the study. GAR, ECJ, FYJ, JFT, EJW and LAC participated in writing and editing to the manuscript. All authors read and approved the final manuscript.
